# The Care of Children's Teeth

**Published:** 1891-11

**Authors:** 


ARTICLE IV.

### THE CARE OF CHILDREN'S TEETH.

**Read before the Southern Dental Association by Dr. Crawford.**

To write something that would be entitled to consideration by this body is an attainment much to be desired; but to write something upon the subject indicated by the caption of my paper, calculated to excite discussion and action on the part of the dental profession and the people of the United States, would be an honor more exalted.

The care of the teeth of children is to day the most important question that addresses itself to the American people. The almost universal presence of disease in the mouths of the children of our common country, is a startling fact that appeals to all who give health and hygiene any attention whatever. I feel certain that some of the statements I may make in reference to this question will subject me to severe criticism at the hands of some, but this amounts to nothing when I feel certain that these statements are correct.

That the teeth of this country are, to a greater or less extent, becoming more susceptible to decay and disease, is to my mind apparent, which fact is more attributable to want of function than any other one thing. Teeth do not only begin to decay earlier in life than was formerly the case, but they continue to succumb to various diseases to a much later period in human existence than they did originally. And that many affections of the teeth are more virulent and rapid in the processes of destruction than they used to be, is a fact that should be given due weight in this discussion.

This position is sustained by the observation that many of our therapeutic agents are losing their value, comparatively speaking. From want of functional activity we find the roots of teeth tardy in their development, so that in many cases extensive decay has occurred before the apical foramen is closed, and the tooth pulp ceased to be a persistent one.

That the presence of large cavities in the teeth, whether filled or not, *does* retard the proper development of the roots in many cases, I feel perfectly certain. Just as the presence of large cavities, and especially large cavities in children's teeth with amalgam fillings in them, retards the normal absorption of the roots of the first twenty prior to their removal; hence, metals should not be used in the treatment of children's teeth, *especially amalgam.* A very small amalgam filling in a child's tooth will, to a greater or less extent, retard if not prevent the physiological process of absorption of the root.

After the above statements by way of explanation of what we have to say in regard to the treatment or management of all cases coming under our care, we will give a few rules that should be enforced by the dental profession:

1. All children should have a competent dentist to superintend the eruption of the first set of teeth.

2. In all cases of infantile trouble, where the diagnosis is doubtful, a dental expert should be called in consultation to determine whether dental irritation is a serious factor or not.

3. All children, after the eruption of the first set of teeth, which usually occurs about the twenty-fourth month, should be subjected to from one to four examinations each year, the frequency of such examinations to be determined by the physiological make-up of each individual case, and give such attention as to prevent and control dental caries, which is about the only affection we have to combat in the management of the first set of teeth, unless gross neglect after the crisis of eruption has passed, has allowed the little sufferers to pass into a state pitiable indeed to behold.

4. No child should be allowed to enter the time of erupting the permanent teeth with caries preying upon all or any of the temporary set, as the fluids of the mouth would be liable to transmit to the second set the germs of disease, or at least all the predisposing conditions to dental caries would be augmented.

5. I would most earnestly insist that all children should have a skillful dentist to conduct the removal of deciduous teeth, *and this should be accomplished in pairs*.

By thus conducting the shedding of the milk teeth, most of the irregularities of the permanent ones would be prevented.

In the treatment of children's teeth, we feel certain that for incipient caries on the proximate surfaces, separating with the Arthur corundum discs, or file to that extent that all small cavities are removed so thoroughly that no trace of disease is left. In cases where the cavities are too deep to admit of removal by the use of the disc, the decay should be reamed out and the cavity filled with some of the various cements, this is to be removed as often as necessary to keep the teeth in a good sanitary condition.

In more advanced cases, where we not only have small cavities on the approximate surfaces, but deep-seated decay in the crowns or grinding surfaces, they should be well prepared and filled with some of the preparations as indicated above. One other class of cases to which I wish to invite attention is, a more advanced and complicated type. Children patients, who present themselves with one or more dead teeth to treat and look after, with or without open sinusses or abscesses, such cases should be treated until a cure is effected, filled and ground down with corundum stones, completely non-antagonizing them so that the non-absorption of their roots may be supplemented by the process of exfoliation. A clinical fact we would do well to note is, that when an abscess has developed as a result of the death of the pulp in a deciduous tooth, the eruption of the permanent one is accomplished much earlier, as a rule, than nature intends they should appear.

The care of children's teeth, or the treatment of the first set, which is composed of twenty, ten in each jaw, has not received that attention at the hands of authors and practitioners that the magnitude and importance of the subject demands. As an humble yet enthusiastic advocate of the claim of our profession to public recognition, I am compelled to say that if dentistry ever takes that high position that it is entitled to, and accomplishes the amount of good that it is capable of accomplishing for humanity, this department of practice must be more closely attended to in the future than it has been in the past.

The fact, that of the scholastic population of our country ninety-five per cent. or a little more, are affected with dental caries, is an appalling statement and bodes more evil to the American people than any other fact, from a healthful standpoint.

If I was asked to make a last statement as to what I believed would be most beneficial to future generations, or to make a last request, and that request be granted, I would state that the application of the art of dentistry to the wants of the *whole* people would do *more* good than any other hygienic procedure that could possibly be instituted, and request that all people, and especially the people of America, be supplied with a sufficient number of good, competent dentists to keep the mouths of at least the children of our country in proper condition.

One of the first things to be looked to in the management of the child, is the undivided and absolute support of the parents or those having charge of the little patients. To attempt to serve a little child whose father and mother is all the time pulling back and questioning the propriety or correctness of the dentist in attempting to keep disease out of the mouth, affords a difficulty that will prevent the most skillful dentist from accomplishing the desired result. Let me say just here, that the fathers and mothers of America are more derelict in their duty in regard to the care of the teeth of their children than in any other particular, and I here state that it is my firm conviction that this dereliction is largely responsible for the large death rate in the children of this country. The startling fact, that fully one-third or a little more of the deaths of this country occur with children under five years of age, is appalling and makes one shudder to contemplate. Hence the emphasis with which we have attempted to call attention to the few points in this short and hurriedly written paper.

In conclusion, allow me to say, while there are some good medicines to be used in the treatment of cases of the young, that most of the relief afforded is by good surgery, based upon proper diagnosis, followed by good sanitation.

In all cases not to cleanse the mouth and teeth after taking food, is a sin against good health and should not be tolerated by good society.

I cannot conclude this short paper without entering my protest against the habit of eating between meals, especially such things as candies and sweetbreads furnished by the little fruit-stands at the street corners and mouths of alleys of our cities. Such places being, in my judgment, a greater menace to public health and welfare than the so much abused whisky traffic in this country.—*Southern Dental Journal.*

